# Late presentation of gastric duplication cyst in pediatric patient: Lessons learned

**DOI:** 10.1016/j.radcr.2024.04.057

**Published:** 2024-05-25

**Authors:** Fatima Chait, Nourrelhouda Bahlouli, Chaymae Faraj, Sara Essetti, Nidal Mrani Alaoui, Narjiss Alami, Bouchra El basri, Naima Erraimi, Zaitouna Alhamany, Nazik Allali, Siham El Haddad, Latifa Chat

**Affiliations:** a Pediatric radiology department, Pediatric Teaching Hospital, Mohammed V University, Rabat Morocco; b Departement of pediatrics, Cheikh Zayd Hospital, Av. Allal Al Fassi, Rabat 10000, Morocco; c Anatomo-pathological department, Cheikh Zayd Hospital, Av. Allal Al Fassi, Rabat 10000, Morocco

**Keywords:** Gastric duplication cyst, Epigastric pain

## Abstract

Gastrointestinal duplication is an infrequent congenital disorder characterized by the presence of a muscular layer covered by mucosa. Gastric duplication cysts account for approximately 2%-9% of all gastrointestinal duplication cysts. The typical clinical presentation often includes symptoms such as epigastric pain, vomiting, and the presence of a palpable abdominal mass. However, these symptoms can overlap with more common conditions. Diagnostic confirmation usually necessitates additional imaging studies, and surgical intervention is the recommended treatment approach. In this case report, we present the case of a 9-year-old girl who presented with chronic abdominal pain and vomiting. Following a comprehensive evaluation, including a CT scan and various diagnostic tests, a diagnosis of gastric duplication cyst was established. The patient subsequently underwent a laparotomy procedure, during which the cyst was completely excised. Follow-up visits indicated an uneventful recovery, with complete resolution of all symptoms. The aim of this work is to report on the clinico-radiological aspects of gastric duplication cysts and their surgical treatment.

## Introduction

Duplication of the alimentary tract is a relatively uncommon congenital anomaly. It can affect any part of the gastrointestinal tract, with the ileum being the most commonly involved site [1,2]. These malformations are believed to be present since birth, formed before the differentiation of the epithelial lining, and therefore named according to the associated organ [3]. Gastric duplication cysts (GDC) account for approximately 2%-9% of all gastrointestinal duplication cysts, with the majority found in the large curvature [4]. Around 67 percent of gastric duplication cysts (GDCs) are typically detected during the initial year of life [5].

Typically, the clinical presentation of GDC is characterized by epigastric pain, vomiting, and the presence of a palpable abdominal mass. Confirming the diagnosis often requires additional imaging tests such as ultrasound, computed tomography, and/or magnetic resonance imaging. Surgical intervention is the preferred treatment approach [1,4].

Due to the rarity of this condition and the limited number of reported cases of GDC, it is crucial to document and report such a case for further understanding.

## Case report

We are reporting the case of a 9-and-a-half-year-old child, the eldest of 2 siblings, born to non-consanguineous parents. The child has been experiencing post-prandial vomiting since the age of 6 months treated as gastrointestinal reflux with slight improvement. Currently, there is a progressive worsening sensation of heaviness accompanied by early satiety even with minimal food intake. Upon examination, a mass is observed in the right hypochondrium.

An abdominal ultrasound showed a thin-walled cystic formation with a pure anechogenic content in the left adrenal region.

**Fig. 1:**
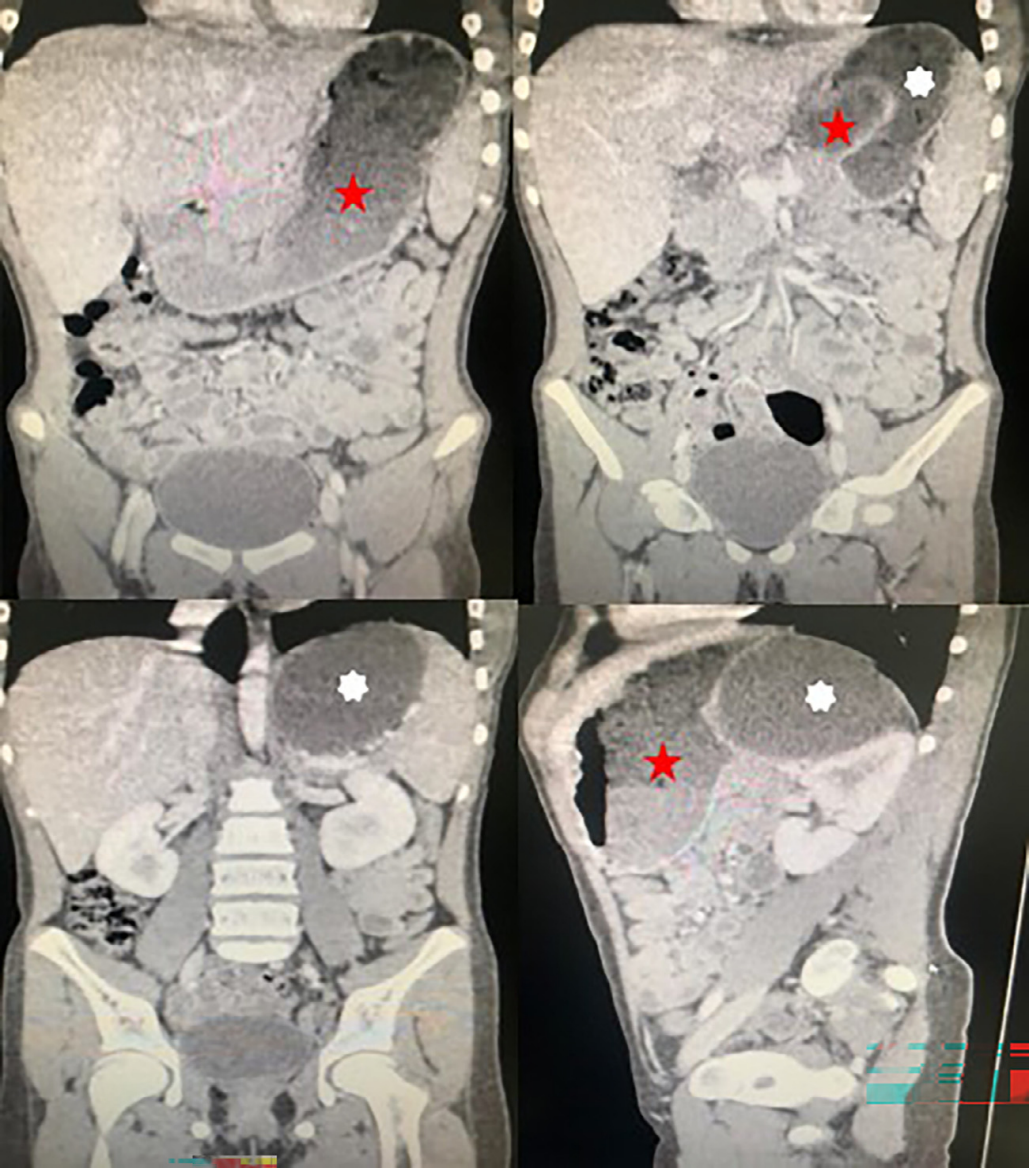
Injected abdomino-pelvic CT images in coronal (A-C ) and sagittal (D) reconstruction showing a non-communicating retro-gastric cystic formation, measuring approximately 87 × 59 mm (White Astérix). Red Star (Stomach).

This was followed by an abdominal CT scan to better characterize the formation, which revealed a retro-gastric cystic mass not communicating with any neighbouring structures, giving rise to the first suggestion of a gastric duplication cyst Fig. 1.

**Fig. 2:**
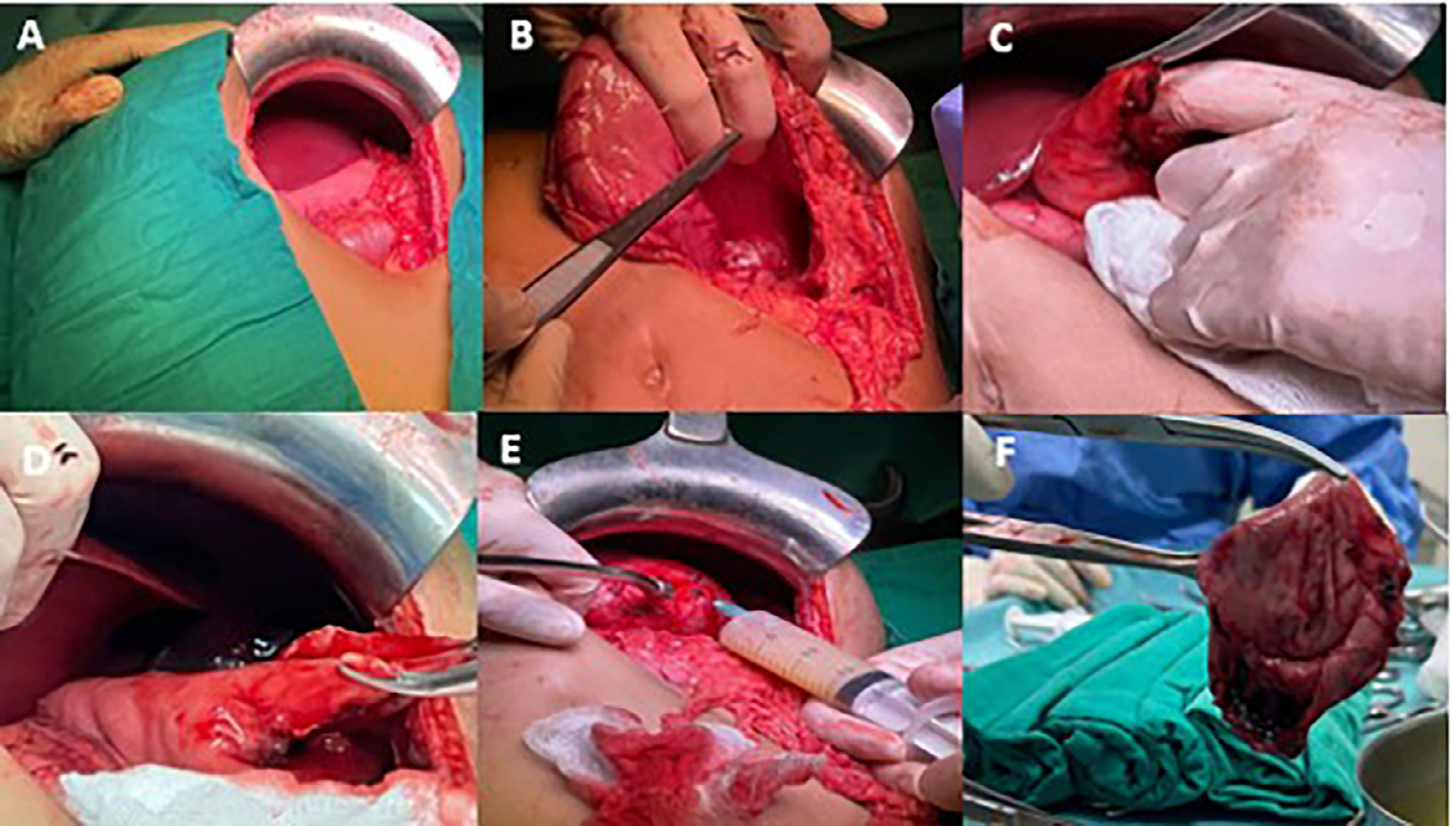
Intraoperative images showing cyst resection steps (Blue star).

A left subcostal transverse surgical approach was utilized, which revealed the presence of a retro-gastric mass located near the greater curvature of the stomach and extending up to the abdominal esophagus. the cyst was punctured and evacuated Fig. 2. Subsequently, a meticulous dissection was carried out, ensuring the complete removal of the mass without causing any harm to the stomach or esophagus Fig. 2.

The postoperative course was uneventful with the resumption of bowel movements on postoperative day 2.

The macroscopic examination of the specimen revealed a cystic pouch weighing 14g, measuring 5.5 × 4.4 cm, corresponding to a highly modified gastric duplication.

**Fig. 3:**
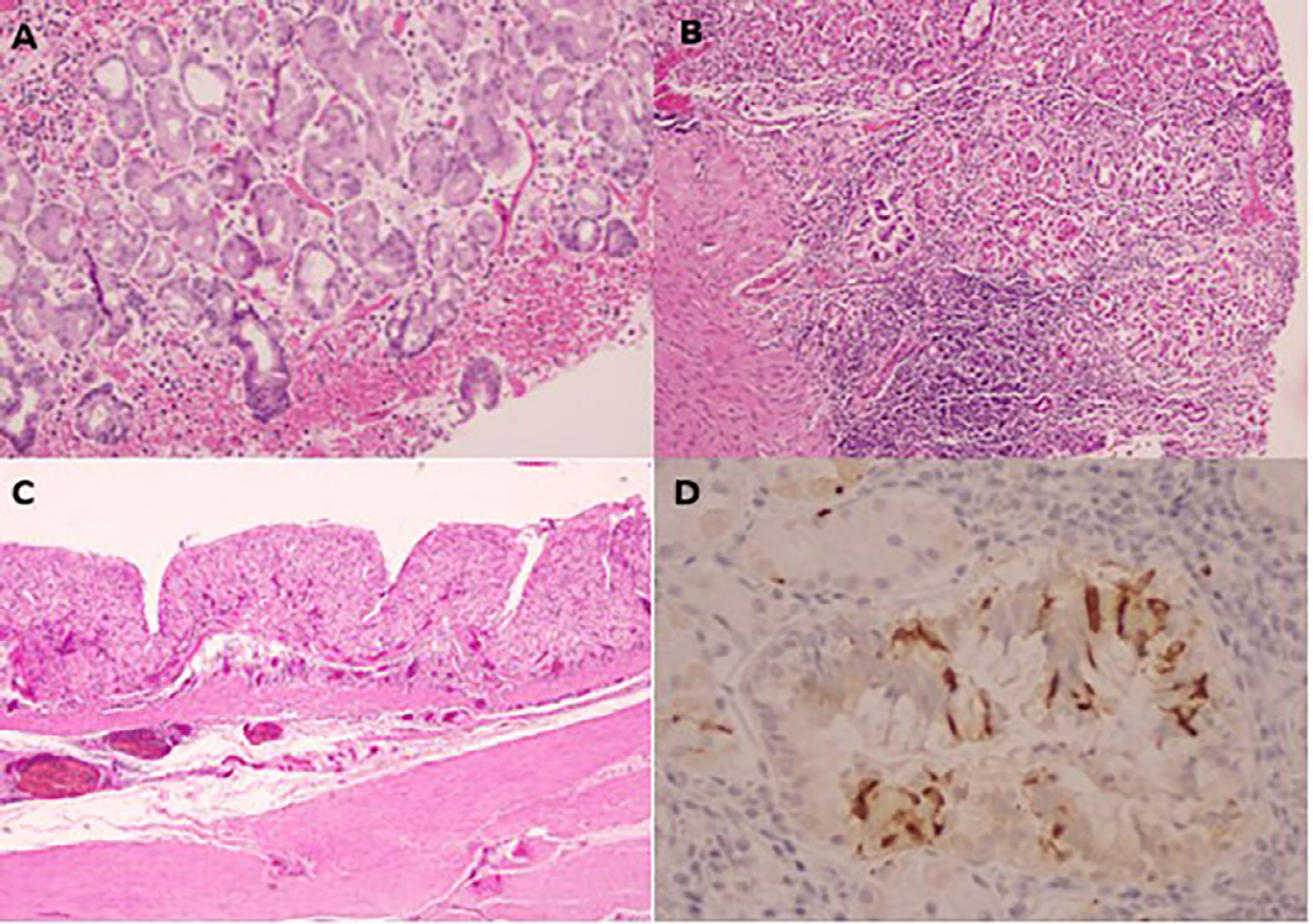
Anatomopathological images showing a fundic-type mucosa (A) with neuroendocrine cells (B) with abrasion and ischemia of a large part of the mucosa replaced by a histiocytic reactionary pseudogranular cell population and an enterochromaffin-like cell population (C and D).

On microscopic examination, the mucosa showed fundic-type features with lesions consistent with minimally active chronic gastritis and positive Helicobacter pylori infection without intestinal metaplasia or dysplasia Fig. 3.

A significant portion of the mucosa exhibited abrasion and ischemia, replaced by a reactive histiocytic population with pseudo-granulomatous cells and a population of enterochromaffin-like cells Fig. 3.

Which confirmed the diagnosis of a gastric duplication cyst.

The child's post-operative outcome was favorable, with no complications.

## Discussion

Gastrointestinal duplication cysts are uncommon birth defects that can occur at any location along the gastrointestinal tract , spanning from the oral cavity to the rectum. They are most frequently found in the ileum (33%), followed by the esophagus (20%), colon (13%), jejunum (10%), stomach (7%), and duodenum (5%) [6].

The incidence of GIDC is approximately 1 in 4500 births, with an occurrence rate of 0.2% among all children. There is a slightly higher prevalence in males compared to females [7].

Gastric duplication cysts are considered rare, and the majority of reported cases have been observed in pediatric patients [1], [2], [3], [4], [5], [6], [7], [8].

Gastrointestinal duplication cysts are rare anomalies that occur during embryonic development, typically between the fourth and eighth weeks. The exact cause of GIDCs remains unknown, and several theories have been proposed to explain their origin and development. These theories include the split notochord theory, luminal recanalization theory, incomplete or partial twinning theory, persistent embryonic diverticula theory, and the intrauterine vascular accident theory. However, none of these theories can fully explain all types of duplications, their locations, and associated anomalies. This suggests that the etiology of GIDCs may be multifactorial [6], [7], [8], [9],10].

Anomalies associated with gastrointestinal duplication cysts such as spinal defects, cardiac abnormalities, and urinary malformations, have been reported at an incidence rate of 16%-26%. Consequently, when an GIDC is diagnosed, it is necessary to conduct further investigations to identify any potential coexisting anomalies [10].

Gastrointestinal duplication cyst possess 3 defining characteristics: an epithelial lining containing gastrointestinal mucosa, a smooth muscle envelope, and close attachment to the gastrointestinal tract through a shared wall. While the mucosal lining of GIDCs may not always match adjacent gastrointestinal tissue, the name of the duplication is based on its attachment site. Ectopic gastric mucosa is present in 20%-30% of GIDCs, particularly in esophageal and small bowel duplications. In some cases [11]. Ectopic pancreatic mucosa is most commonly associated with gastric duplications.

The clinical presentation of gastric duplication cysts can vary greatly and lack specificity. Symptoms range from simple abdominal pain to nausea, vomiting, along with abdominal tenderness and the presence of an epigastric mass during physical examination [12]. These cysts have the potential to compress neighboring organs such as the pancreas, kidneys, spleen, and adrenal glands, leading to a differential diagnosis that includes lesions originating from these organs [2]. Complications associated with duplication cysts may include infection, ulceration, hemorrhage, perforation, fistula formation, obstruction, compression, or malignant transformation [13].

Diagnostic investigation for gastric duplication cysts includes ultrasound, computed tomography (CT), and magnetic resonance imaging (MRI) [14]. Gastric duplication cysts can pose a diagnostic challenge as they may be confused with pancreatic cysts, adrenal cysts, or stomach adenomas. If a patient presents with symptoms such as hematemesis or vomiting, accompanied by a palpable abdominal mass, a CT scan is recommended to obtain a more definitive diagnosis. The definitive diagnosis of gastric duplication cysts is typically established through surgery and subsequent histopathological examination [14,15].

To prevent potential complications such as obstruction, torsion, perforation, hemorrhage, and malignancy [12], the preferred therapeutic approach is complete excision of the gastric duplication cyst. Complete excision is the standard treatment for a communicating cyst [13]. When both gastric lumens are patent, communicating gastric duplication generally does not require intervention 13]. Although drainage and marsupialization of the cyst have been suggested, marsupialization in the stomach exposes the vulnerable cyst mucosa to gastric contents, increasing the risk of ulceration [5]. Drainage procedures like cystojejunostomy can lead to complications such as anastomotic stenosis or blind loop syndrome and are therefore not recommended. Furthermore, leaving the cyst in place is not advisable due to the risk of malignant transformation [5].

The preferred approach for treating gastrointestinal duplications involves thorough removal, typically including adjacent intestines and mesentery. However, in cases of completely isolated duplication cysts, it can be safely performed without necessitating bowel resection. Recognizing this type of duplication cyst informs surgeons in selecting the most appropriate surgical procedure. For asymptomatic, enlarging intra-abdominal cystic masses with a preliminary diagnosis of duplication cysts, laparoscopic exploration should be considered as the primary surgical option. The benefits of laparoscopy can significantly enhance patient care [16].

The primary differential diagnoses for gastric duplication cysts include lymphangiomas, pancreatic pseudocysts, and mesenteric cysts [17].

Duplication cysts in the alimentary tract are frequently observed in children, necessitating surgical resection in most instances. While a subset of cases may demand intricate surgical procedures, reports indicate excellent long-term outcomes following optimal surgery.

## Conclusion

Gastrointestinal duplication is a rare congenital disorder, and gastric duplication is even more uncommon, with fewer than 50 reported cases. The clinical picture is non-specific, which often leads to late diagnosis.

A thorough clinical examination, combined with additional investigations, plays a crucial role in establishing an accurate diagnosis. This facilitates the implementation of appropriate treatment, which often involves complete surgical removal of the lesion whenever feasible. This approach aims to prevent potential complications in the future, including the development of malignancy.

## Ethical approval

Our institution does not require ethical approval for reporting individual cases or case series.

## Patient consent

Written informed consent was obtained from the parents of patients for the publication of this case report.

## Guarantor of submission

The corresponding author is the guarantor of submission.

## Author contributions

**FC:** Contributed to conception and design; Contributed to analysis; Drafted the manuscript; Gave final approval; Agrees to be accountable for all aspects of work ensuring integrity and accuracy.

**NB , CF and SE:** Contributed to conception and design; Contributed to analysis; Agrees to be accountable for all aspects of work ensuring integrity and accuracy.

**NMA**: Critically revised the manuscript; Gave final approval; Agrees to be accountable for all aspects of work ensuring integrity and accuracy.

**NA, BE,NE:** Contributed to conception and design; Contributed to analysis; Agrees to be accountable for all aspects of work ensuring integrity and accuracy

**ZA, NA and SEH:** critically revised the manuscript; Gave final approval; Agrees to be accountable for all aspects of work ensuring integrity and accuracy. **LC**: Contributed to acquisition, analysis, or interpretation; Critically revised the manuscript; Gave final approval.
